# Tie2/TEK Modulates the Interaction of Glioma and Brain Tumor Stem Cells with Endothelial Cells and Promotes an Invasive Phenotype

**DOI:** 10.18632/oncotarget.204

**Published:** 2010-12-30

**Authors:** Dan Liu, Vanesa Martin, Juan Fueyo, Ok-Hee Lee, Jing Xu, Nahir Cortes-Santiago, Marta M. Alonso, Kenneth Aldape, Howard Colman, Candelaria Gomez-Manzano

**Affiliations:** ^1^Department of Neuro-Oncology, The University of Texas M. D. Anderson Cancer Center, Houston, Texas, USA; ^2^Severance Hospital Integrative Research Institute for Cerebral and Cardiovascular Disease, Yonsei University Health System, Seoul, Korea; ^3^Department of Pathology, The University of Texas M. D. Anderson Cancer Center, Houston, TX, USA; ^4^Department of Genetics, The University of Texas M. D. Anderson Cancer Center, Houston, TX, USA

**Keywords:** Tie2/TEK, glioma, endothelial cells, invasion, microenvironment

## Abstract

Malignant gliomas are the prototype of highly infiltrative tumors and this characteristic is the main factor for the inevitable tumor recurrence and short survival after most aggressive therapies. The aberrant communication between glioma cells and tumor microenvironment represents one of the major factors regulating brain tumor dispersal. Our group has previously reported that the tyrosine kinase receptor Tie2/TEK is expressed in glioma cells and brain tumor stem cells and is associated with the malignant progression of these tumors. In this study, we sought to determine whether the angiopoietin 1 (Ang1)/Tie2 axis regulates crosstalk between glioma cells and endothelial cells. We found that Ang1 enhanced the adhesion of Tie2-expressing glioma and brain tumor stem cells to endothelial cells. Conversely, specific small interfering RNA (siRNA) knockdown of Tie2 expression inhibited the adhesion capability of glioma cells. Tie2 activation induced integrin β1 and N-cadherin upregulation, and neutralizing antibodies against these molecules inhibited the adhesion of Tie2-positive glioma cells to endothelial cells. In 2D and 3D cultures, we observed that Ang1/Tie2 axis activation was related to increased glioma cell invasion, which was inhibited by using Tie2 siRNA. Importantly, intracranial co-implantation of Tie2-positive glioma cells and endothelial cells in a mouse model resulted in diffusely invasive tumors with cell clusters surrounding glomeruloid vessels mimicking a tumoral niche distribution. Collectively, our results provide new information about the Tie2 signaling in glioma cells that regulates the cross-talk between glioma cells and tumor microenvironment, envisioning Tie2 as a multi-compartmental target for glioma therapy.

## INTRODUCTION

Tumor microenvironment plays an important role in tumor progression and invasion. Interaction of tumor cells with the extracellular matrix and stromal cells is crucial for tumor formation including survival, invasion and maintenance of tumor-initiating cells [1]. Malignant gliomas are the most common type of primary brain tumor, and the prognosis for patients with these tumors remains dismal [2]. Diffuse, infiltrative invasion of glioma cells into surrounding brain tissue results in tumor recurrence and contributes to the poor prognosis of patients with malignant gliomas [3]. Understanding the molecular mechanisms that underlie tumor glioma progression and local invasion of gliomas represents one of the great challenges in exploratory cancer research.

The tyrosine kinase receptor Tie2/TEK was initially reported as a specific vascular receptor present in both normal and tumoral endothelial cells (EC), including ECs in astrocytomas, and its levels correlate positively with increasing malignancy [4-8]. In these processes, Tie2 and its ligands, angiopoietins, are critical to angiogenic remodeling [9]. In addition, Tie2 was found in hematopoietic stem cells and its function was reported as required for postnatal bone marrow hematopoiesis and protecting the hematopoietic stem cell compartment from myelosuppressive stress [10,11]. We previously showed that Tie2 is expressed in glioma cells and brain tumor stem cells present in malignant gliomas [12,13]. In the current study, we investigated whether the Angiopoietin 1 (Ang1)/Tie2 axis regulates the crosstalk between glioma cells and the tumor microenvironment. Examining in vitro and in vivo models of gliomas, we found that glial Tie2 is involved in enhancing the adhesion of glioma cells to ECs, and critically participates in the development of the invasive phenotype of gliomas.

## RESULTS

### Tie2 Activation Enhances the Adhesion of Glioma Cells to Endothelial Cells

**Figure 1:**
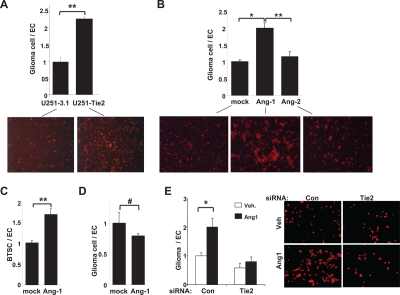
Tie2 activation increases the adhesion of glioma cells to ECs (A) The adhesion of U251.Tie2 cells to HUVECs was significantly higher than that of U251.vector cells to HUVECs. Data are presented as the relative ratio of the adhesion of glioma cells to ECs (adhesion of U251.vector cells to ECs equals to 1). Fluorescence microphotographs of glioma-HUVEC co-cultures following the removal of non-adherent glioma cells are also shown (PKH26 staining; × 50). (B and C) The adhesion of U-87 MG glioma cells (B) and GSC20 BTSCs (C) to HUVECs after Ang1 or Ang2 treatment. Data are the relative ratio of the adhesion of glioma cells to ECs (adhesion of untreated glioma cells to ECs equals to 1). Fluorescence microphotographs of glioma-HUVEC co-cultures taken following the removal of non-adherent glioma cells are also shown (*B*; PKH26 staining;x 25). (D) Adhesion studies of Tie2-negative U-373 MG cells after Ang1 treatment. Data are the relative ratio of the adhesion of glioma cells to ECs (adhesion of untreated glioma cells to ECs equals to 1). Data in each panel are the means ± SD from three independent experiments performed in triplicate. *, *P* < .05; **, *P* < .001; #, *P* > .05. *E*, U-87 MG cells were transfected with Tie2 siRNA or control siRNA and exposed to Ang1 before adhesion assay. Data are the relative ratio of the adhesion of glioma cells to ECs (adhesion of U-87 MG cells transfected with control siRNA and exposed to vehicle equals to 1). The means ± SD from three independent experiments performed in triplicate are shown. *, *P* < 0.05. Fluorescence microphotographs show the adhesion of U-87 MG glioma cells to endothelial cultures after the indicated treatments (PKH26 staining; original magnification × 100).

To characterize the role of Tie2 activation in glioma cell–EC interaction, we used a co-culture system in which the adhesion of fluorescent dye–labeled glioma cells to an EC monolayer was quantified by flow cytometry. We found that the adhesion of U251.Tie2 cells to ECs was more than two-fold higher than the adhesion of U251.vector cells to ECs (Figure 1*A*). We further analyzed the adhesion properties of endogenous Tie2-expressing cells, U-87 MG glioma cells and GSC20 BTSCs [13]. Ang1 significantly stimulated adhesion of both Tie2-expressing glioma cells and BTSCs to ECs (Figures 1*B* and *C*). Of importance, similar experiment performed in the presence of Ang2, a context-dependent Tie2 ligand, did not modify the adhesive properties of glioma cells to ECs (Figure 1*B*).

We tested the specificity of action of Ang1 on Tie2 receptor by performing two different experiments. First, treating Tie2-negative U-373 MG cells [13] with Ang1 did not modify their adhesion to ECs (Figure 1*D*). Second, treatment with Tie2 siRNA significantly abolished Ang1-induced adhesion of U-87 MG cells to ECs (Figure 1*E*). These results indicate that the Ang1/Tie2 axis plays a major role in mediating the adhesion of glioma cells and BTSCs to ECs.

### Tie2 Enhances Adhesion of Glioma Cells to Endothelial Cells by upregulating integrin 1 and N-Cadherin

**Figure 2:**
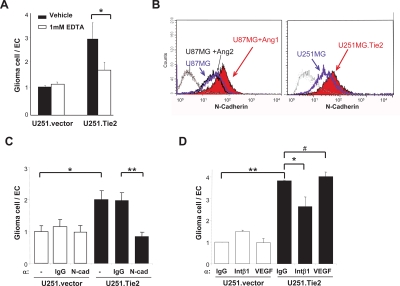
Tie2 activation results in upregulation of adhesion molecules in gliomas (A) The adhesion of U251.vector and U251.Tie2 cells to ECs was performed in the presence of EDTA. *, *P* < .05. (B) The Ang1/Tie2 axis regulates N-cadherin expression. U-87 MG cells treated with Ang1, Ang2, or vehicle, and isogenic U251.vector and U251.Tie2 cells were immunostained with anti-human N-cadherin antibody and analyzed with flow cytometry. The results from one of three independent experiments are shown. Leftmost diagram represents cells stained with isotype immunoglobulin G (IgG). (C) The adhesion of U251.Tie2 cells or U251.vector cells to ECs was performed in the presence of neutralizing antibodies against N-cadherin (C) or integrin β1 (D). IgG was used as a control, and antibodies against VEGF were used for specificity. Data are the relative ratio of the adhesion of glioma cells to ECs (adhesion of U251.vector cells to ECs in the presence of mock (C) or IgG (D) equals to 1). The means ± SD from three independent experiments performed in triplicate are shown. *, *P* < .05, **; *P* < .001; #, *P* > .05.

To determine which molecules are involved in Tie2-mediated adhesion of glioma cells to ECs, we performed cell adhesion experiments in the presence of EDTA and found a significant decrease in the number of Tie2-positive glioma cells that adhered to EC monolayers, suggesting the contribution of calcium-dependent adhesion molecules (Figure 2*A*). Because cell-to-cell adhesion involves the interaction of adhesion molecules on the cell surface, we then used flow cytometry to analyze the cell-surface expression of N-cadherin. U251.Tie2 cells expressed significantly higher levels of N-cadherin than U251.vector cells (Figure 2*B*). Similarly, U-87 MG cells expressed higher levels of surface N-cadherin after Ang1 treatment (Figure 2*B*), as confirmed by Western blot analysis (Supplementary Figure 1). However, treating U-87 cells with Ang2 did not significantly modify the expression of cell-surface N-cadherin (Figure 2*B*). We used quantitative RT-PCR to compare the mRNA levels of N-cadherin, E-cadherin, α-catenin, and β-catenin in U251.vector versus U251.Tie2 cells and U-87 MG cells after treatment with Ang1 or vehicle. We did not observe any significant modification in the mRNA levels of N-cadherin, α-catenin, or β-catenin (E-cadherin expression was not detectable) in relation to Tie2 activation, suggesting a post-transcriptional regulation of these molecules (Supplementary Figure 2).

To determine whether N-cadherin is involved in Tie2-mediated glioma cell adhesion, we incubated U251.Tie2 cells with a neutralizing antibody against N-cadherin before and during their co-culture with ECs. We found that the adhesion of U251.Tie2 cells to ECs in the presence of neutralizing antibodies against N-cadherin completely abolished the Tie2-mediated adhesion to ECs (Figure 2*C*). To determine whether the upregulation of integrin ß1 is involved in Tie2-mediated glioma cell-to-EC adhesion, we performed adhesion experiments in the presence of an integrin ß1–neutralizing antibody. We observed a significant decrease in the adhesion of U251.Tie2 glioma cells to ECs. Suggesting specificity of these results, a neutralizing antibody for VEGF, a molecule involved in cell-to-cell interaction [14], did not modify the adhesive properties of Tie2-expressing glioma cells (Figure 2*D*). These data suggested that both N-cadherin and integrin ß1 play major roles in the Tie2-mediated adhesion of glioma cells to ECs.

### Tie2 Activation Stimulates Glioma Cell Invasion in Vitro

**Figure 3:**
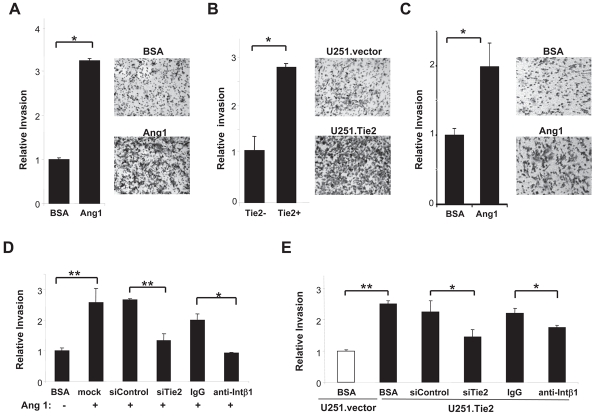
Tie2 activation enhanced the invasive properties of glioma cells (A-C) Invasion assays were performed in Tie2-expressing U-87 MG cells (A) and NSC20 BTSCs upon Ang1 exposure (C) and in isogenic-derived U-251 MG cells (B). Data are the relative ratio of invasive glioma cells with active Tie2 compared to invasive vehicle-treated glioma cells (A and C) or U251.vector cells (B) (equal to 1). The means ± SD from three independent experiments performed in triplicate are shown. Representative microphotographs show glioma cells invading Matrigel under the indicated culture conditions (original magnification × 50). Tie2 expression was downregulated by transfecting the cells with Tie2 siRNA before invasion assay. *, *P* < .05, **, *P* < .01.

Because our data suggest that Tie2 activation increased cell adhesion properties and N-cadherin and integrin ß1 expression, and alterations in these two molecules have been implicated in the invasive phenotype of malignant gliomas [15,16], we next sought to determine whether Tie2 activation modulates glioma cell invasion. Invasion assay revealed that the number of U251.Tie2 cells that migrated through the Matrigel was more than 3-fold the number of U251.vector cells that migrated through the Matrigel (Figure 3*A*). Similarly, cells expressing endogenous Tie2 showed a higher degree of invasion when treated with Ang1. Thus, Ang1-treated U-87 MG cells invaded the basement membrane about three-fold more than vehicle-treated U-87 MG cells. These results were confirmed by studying the role of Tie2-positive BTSCs, GSC20, in invasion. Thus, the number of GSC20 cells migrated was two-fold higher when the cultures were exposed to Ang1 than to the vehicle. Together these data suggested a role of Ang1/Tie2 in invasion of glioma cells.

To further clarify the role of Tie2 in the invasive phenotype, we used specific siRNA to knock down Tie2 expression. Tie2 downmodulation resulted in the partial rescue of the invasive phenotype in both Ang1-treated U-87 MG cells (Figure 3*D*) and U251.Tie2 cells (Figure 3*E*). Moreover, neutralizing antibodies against integrin β1 inhibited the Tie2-mediated invasion of both glioma cell lines. These data suggest that the Ang1/Tie2/integrin β1 axis contributes to glioma cell invasion *in vitro*.

**Figure 4:**
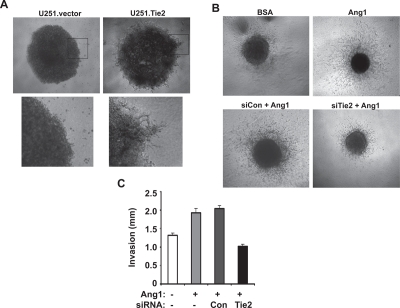
Tie2 activation increase tumor cell invasion of 3D matrix (A) Cell spheroids generated from U251.vector or U251.Tie2 cells invaded collagen-enriched Matrigel after 11 days of culture. Phase contrast pictures showed the invasive front of these spheroids (close-up of the boxed areas). (B) U-87 MG cell spheroids were cultured in 3D collagen gel with or without incubation of 500ng/ml Ang1 (upper panel). U-87 MG cell were also treated with control or Tie2 siRNA before incorporated into 3D collagen gel and incubation of Ang1 (lower panel). Phase contrast pictures showed cell invasion of collagen matrix on day 3. The distance from invasive front to the edge of U-87 MG cell spheroids was measured and compared in different treatment groups (C).

To better mimic the in situ tumor confining and allow the contact with 3D matrix, we generated 3D cultures of U251.vector and U251.Tie2 cells in an enriched collagen I matrix since collagen I is one of the major components of the extracellular matrix of gliomas and the activation of Tie2 resulted in increased adhesion to this extracellular component [12]. We observed that the 3D U251.Tie2 spheroids acquired an invasive phenotype consisting on a single-cell migration pattern from the periphery of the spheroids. This characteristic pattern was observed as soon as 72 hrs of the initiation of the 3D culture and was maintained by 11 days, the last time point examined. However, cells from 3D U251.vector spheroids did not displayed significant migration through the matrix (Figure 4*A*). Confirmatory experiments were performed using U-87 MG-derived spheroids. U-87 MG cells were pre-incubated with Ang1 and then spheroids were cultured in a collagen I-enriched matrix containing Ang1. We observed an accelerated rate of migration of Ang1-stimulated U-87 MG cells through the matrix. This response was partially abrogated by knocking-down Tie2 expression (Figures 4*B* and *C*). These results corroborated an essential role of Tie2 in brain tumor dispersal *in vitro*.

### Glioma Tie2 Expression Increases Tumorigenesis Characterized by an Invasive Phenotype in Vivo

**Figure 5:**
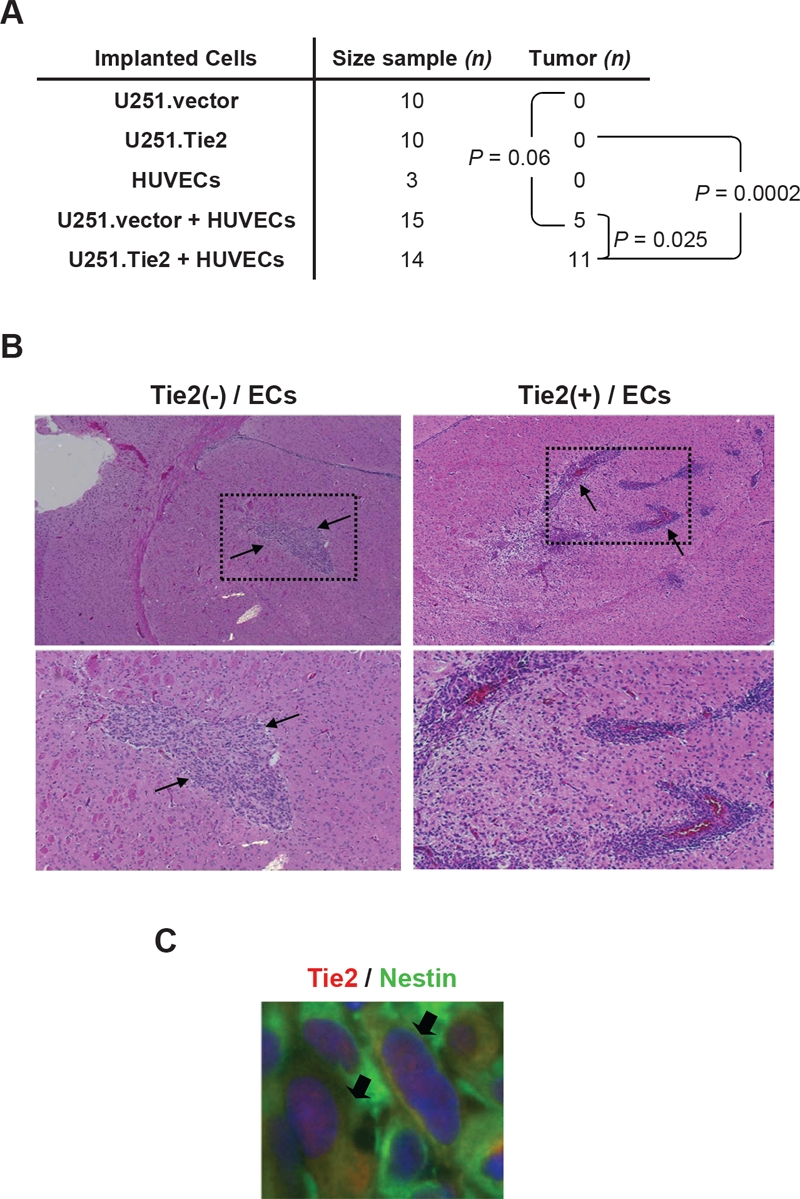
Role of the interaction of Tie2+ glioma cells with ECs in gliomagenesis in vivo (A) Table showing the number of mice injected with the indicated cells and the incidence of tumor formation. Data are the combined results of two independent experiments. *P* values for the comparison of tumor incidence are shown (F fisher test). (B) U251.vector or U251.Tie2 cells were injected alone or with ECs into the brains of immunocompromised mice. Hematoxylin and eosin–stained sections of the resulting xenografts are shown. Black arrows indicate the approximate limits of the tumors (*upper panels*; × 100). Note the infiltrative/multifocal component of tumors derived from Tie2-positive cells injected with ECs. Close-up of the boxed areas in *lower panels*. (C) Tie2 and Nestin double immunostaining performed in a GSC20-derived intracranial xenograft sample. Arrows indicate Tie2 and Nestin co-localization.

To determine whether Tie2-mediated changes in glioma phenotype modulates the neoplastic characteristics of these tumors *in vivo*, we injected U251.vector or U251.Tie2 cells into the brains of immunodeficient mice, and then we sacrificed the animals approximately 20 days after cell implantation, and analyzed their brains for the presence of tumors. Although we observed an increase of tumor formation with co-injection of U251.vector cells with ECs (33%), this number was significantly higher when we co-injected the mice with U251.Tie2 cells and ECs: 11 of 14 (79%) animals developed intracranial tumors (Figure 5*A*). Intriguingly, U251.Tie2 and EC–derived tumors had different features than U251.vector and EC–derived tumors (Figure 5*B*). Thus, glioma cells migrated farther from the site of injection, resulting in multifocal tumors that surrounded vascular structures. Furthermore, and as a novel finding, this pro-tumorigenic force is enhanced and modulated towards an invasive phenotype in the presence of neoplastic glial cells expressing Tie2. To further characterize the glioma Tie2+ cells, we analyzed a BTSC-derived intracranial xenografts. In this animal model, Tie2 positive cells co-stained positive for Nestin expression (30% ± 8% of Nestin+ cells) suggesting a precursor origin (Figure 5*C*) [17]. Together our data suggest that Tie2 plays a non-previously described key role in the crosstalk between the neoplastic glial cells and the vascular compartment that, ultimately, modulates the invasive properties of those tumors.

## DISCUSSION

In this report we provide new information on the role of Tie2 in glioma and brain tumor stem cells. Our data showed that Tie2 activity in glioma cells and BTSCs was related to enhanced adhesion to the endothelial compartment with subsequent increase of their invasion capability. We also found that injecting Tie2-positive cells together with ECs into the brains of immunocompromised animals resulted in the development of invasive, multifocal tumors that surrounded vascular structures. These data are relevant because the invasive phenotype of GBMs is one of the main underlying cause of their refractoriness to conventional treatment and responsible for the invariable recurrence after therapy [18].

Our study is continuation of our previous work on the Tie2-mediated regulation of integrin β1 and the adhesion to several components of the extracellular matrix [12]. Integrin-mediated cell substratum adhesion and migration often occur simultaneously with cadherin-based cell-to-cell adhesion [19-22]. In gliomas, integrin 1 has been reported to be involved in adhesion, migration, and invasion [23, 24]. Moreover, N-cadherin expression levels have been reported to be related to the progression of the malignant phenotype [25]. Here we found that Tie2 activity upregulates N-cadherin in the membrane of glioma cells that function to effectively mediate the cellular interactions with the tumor microenvironment. Several lines of evidence support an orchestrated regulation between cadherin- and integrin-mediated systems [19, 26]. These cellular interactions should occur following a temporal and spatial regulation of cell-to-cell and cell-to-substrate adhesion properties. The fact that Tie2 activation results in cell-to-cell, as well as cell-to-substrate adhesion, strongly suggests that Tie2 signaling is one of the key interactors in the tight and coordinated regulation of these two processes.

Implications of the existence of BTSCs [17, 27] include that these cells are associated with the vasculature in brain tumors constituting the so-called perivascular niche [28]. A corollary of this tenet is the hypothesis that interaction of BTSCs with ECs mediates chemoresistance of cancer cells. Although the interaction of BTSCs with ECs has been implicated in maintaining the self-renewing and undifferentiated state of BTSCs [28], little is known about the mechanism of this critical crosstalk. In the current study, we found that Ang1 significantly enhanced the adhesion of Tie2-positive BTSCs to ECs. These results together with our earlier findings regarding the contribution of Tie2 signaling to the chemoresistance of BTSCs [13, 29] might indicate that Tie2 interacts with BTSCs and contributes to the perivascular, chemoresistant niche. Further studies should ascertain to which extent the roles of Tie2 in mediating BTSC adhesion to ECs and increasing chemoresistance are independent or interdependent signaling pathways.

Preventing gliomas from developing an invasive phenotype is one of the main challenges involved in blocking glioma recurrence and improving outcome. In the current study, we observed that injecting Tie2-positive glioma cells together with ECs resulted in the formation of more tumors that, in turn, tended to be more aggressive than Tie2-negative tumors. Thus, although the role of the tumor microenvironment in modulating the neoplastic phenotype has been previously reported [28], the molecular mechanisms regulating this relationship are still under investigation. The work presented here demonstrates the key role of Tie2 in the influence of the tumor/microenvironment cross-talking on the malignant phenotype of gliomas.

In the fullness of time, our data may have important overtones for the therapy of malignant gliomas. Placing Tie2 as a landscape regulator and therefore, as one of the main interactor in the complexity of the tumor microenvironment regulation, suggests that therapeutic targeting of Tie2 would be a potent anti-multicompartment approach.

## MATERIALS AND METHODS

### Cell Culture

U-87 MG and U-251 MG human glioma cell lines (American Type Culture Collection) were maintained as previously described [12]. Human Umbilical Vein Endothelial Cells (HUVECs; Clonetics) were used before passage 10 and maintained according to the provider's instructions. NSC20/GSC20 neurospheroid cultures were established from a human glioblastoma multiforme (GBM) surgical specimen and maintained as described previously [17]. Tie2-overexpressing U-251 MG (U251.Tie2) cells and vector-transfected U-251 MG (U251.vector) cells were previously generated [12]. We purchased Ang1 and Ang2 from R&D Systems.

### Small Interfering RNA Oligonucleotide Transfection

Human glioma cells were plated in 60-mm dishes (3 × 10^5^ cells per dish). After 18 hours, we used Lipofectamine 2000 (Invitrogen) to transfect the cells with 40 nM Tie2 small interfering RNA (siRNA) or scrambled siRNA oligonucleotides (Santa Cruz Biotechnology) according to Invitrogen's instructions as previously reported and validated [12-13].

### Glioma-to-Endothelial Cell Adhesion Assay

HUVECs were seeded onto 24-well plates (1 × 10^5^ cells/well) 48 hours before adhesion assay. Glioma cells were labeled with PKH26 red dye (2 × 10^−6^ M; Sigma) according to the manufacturer's instructions, resuspended in serum-free medium, and seeded onto HUVEC monolayers at a 5:1 glioma cell–to–EC ratio, and incubated at 37°C. After 15 minutes, we removed non-adherent glioma cells and used fluorescence microscopy (Axiovert 200; Carl Zeiss MicroImaging Inc) to visualize the adhesion of glioma cells to ECs. We then used a 0.05% trypsin/0.02% EDTA (w/v) solution to disrupt aggregates and provide a single-cell suspension for glioma cell quantification by flow cytometry (FACS Calibur; Becton Dickinson). Data were processed using CellQuest Pro software (BD Biosciences).

For calcium-chelating experiments, we pre-incubated glioma cells with 1 mM EDTA for 20 minutes and we added 1mM neutralizing antibodies for 15 minutes before and during co-culture with ECs: anti-N-cadherin (clone GC4; 1:30 dilution; Sigma), anti-integrin ß1 antibody (clone 6S6; 10 µg/ml; Chemicon International), and anti-human VEGF antibody (MAB293; 1µg/ml; R&D Systems). Mouse IgG_1_ (MAB002) or IgG_2B_ isotypes (MAB004; R&D Systems) were used as controls.

### Flow Cytometric Analysis

We used flow cytometry (FACSCalibur) to measure cell-surface N-cadherin expression. We used 5 mM EDTA to detach 1 × 10^6^ glioma cells and incubated the cells with mouse anti-human N-cadherin antibody (clone GC4; 1:900 dilution; Sigma), or the same amount of mouse IgG_1_ (R&D Systems) as negative control at 4°C for 1 hour and with Alexa 488–conjugated anti-mouse antibody (1:100 dilution; Invitrogen) for 15 minutes at room temperature.

### Matrigel Invasion Assay

For invasion assay, we coated transwells (ThinCert TC Inserts, 8.0-µm pore size; USA Scientific) in 24-well plates with 0.3 mg/ml Matrigel (BD Biosciences) and allowed them to adjust to room temperature for 2 hours. Glioma cells (1 × 10^5^) were washed with serum-free medium, suspended in 250 µl serum-free medium, and seeded onto the Matrigel-coated transwells. 600 µl DMEM/F12 supplemented with 2% fetal bovine serum was added to the lower chambers. When indicated, cells were pre-incubated with a neutralizing antibody against integrin ß1 (clone 6S6; 10 µg/ml; Chemicon International), or IgG_1_ as isotype control (R&D System) for 20 minutes at 37°C. Cells were then plated in the transwells in the presence of anti-integrin ß1 antibody. After incubating overnight, cells on the top of the inserts were removed. Inserts were then fixed with methanol and stained with 0.5% crystal violet (Sigma-Aldrich) for 10 minutes, and relative number of cells was quantified by measuring absorbance at 570 nm.

### 3D glioma cultures and invasion assay

To generate glioma cell spheres, 1×10^4^ glioma cells suspended in 100µl DMEM/F12 medium were seeded into a 96-well plate coated with 1% agarose gel and incubated overnight. 96-well plates were coated with collagen-enriched Matrigel, which consisted on mixing on ice the neutralized collagen I (R&D systems) and growth factor reduced Matrigel (BD Bioscience) such that the final collagen concentration was 1.6 mg/ml. Then, the wells were incubated for 20 minutes in 37°C for gelling. Glioma cell spheres were transferred into these collagen-enriched Matrigel-coated 96-well plates, and subsequently overlaid with another layer of 50µl neutralized collagen. After solidification of the upper layer for 15 minutes, 3D cultures were incubated with 150µl serum-free DMEM/F12 medium. When indicated, 500ng/ml Ang1 was both incorporated into the collagen solution before gelling and supplemented into the culture medium. For assessment of invasion, we periodically analyzed by microscopy the presence of invading individual cells and we measured the major invasive front from the spheroid (completely surrounded by the matrix and not in contact with the walls of the well at any point).

### Animal Studies

Animal studies were performed in veterinary facilities at The University of Texas M. D. Anderson Cancer Center in accordance with institutional guidelines. To study whether Tie2 activation in crosstalk with the ECs impacts tumorigenicity, we injected 1 × 10^6^ U251.vector or U251.Tie2 cells alone or in a suspension containing 2 × 10^5^ HUVECs into the caudate nuclei of 4- to 6-week-old female athymic mice (Harlan-Sprague Dawley, Inc.) as previously described [30]. To study Tie2 expression in brain tumor stem cell (BTSC)-derived intracranial xenografts, we implanted 5 × 10^5^ GSC20 cells into the caudate nuclei of 4- to 6-week-old female athymic mice and sacrificed the animals when showing generalized or localized symptoms of toxicity were sacrificed. We removed the brains, fixed in 4% formalin, and embedded them in paraffin. Hematoxylin and eosin–stained coronal sections were evaluated for evidence and characterization tumors.

### Immunohistochemistry and Immunofluorescence Studies

For double immunofluorescence studies, tissue sections were blocked with 10% goat serum following heat-induced antigen retrieval and then incubated with anti-Tie2 antibody (C-20; 1:50; Santa Cruz Biotechnology) and anti-Nestin (MAB5326; 1:200, Chemicon International). Texas red– and fluorescein isothiocyanate–conjugated secondary antibodies (Santa Cruz Biotechnology), respectively, were used for 1 hour at room temperature.

### Statistical Analyses

For quantitative data analysis, the results were plotted as the mean ± SEM. Statistical analysis was performed using GraphPad Prism version 3.0a (GraphPad Software Inc.). Statistical significance was determined using Student's t tests, or for multiple comparisons using two-way analysis of variance (ANOVA). For enumeration data analysis, the results were plotted as the frequencies. Statistical significance between groups was determined using chi-square analysis.

## SUPPLEMENTAL FIGURES

Supplemental Figure 1 Tie2-mediated modulation of adhesive molecules in gliomas at protein levels. Western blotting analysis of U-87 MG cells treated with Ang1 or vehicle and of U251. vector and U251. Tie2 showing the expression level of N-cadherin and Integrin β1. Tubulin is shown as loading control.

Supplemental Figure 2 Tie2-mediated modulation of adhesive molecules in gliomas at transcriptional levels. qPCR analysis of (*A*) U251.vector and U251.Tie2 and (*B*) U-87 MG cells treated with Ang1 or vehicle showing the expression level of N-cadherin, α-catenin, and β-catenin. Results represent ΔC_T_ levels (mean ± SEM) over endogenous control GAPDH.
