# Supplementary material for: Tie2/TEK Modulates the Interaction of Glioma and Brain Tumor Stem Cells with Endothelial Cells and Promotes an Invasive Phenotype
Source: Oncotarget. 2010 Dec 30;1(8):700–9. doi: 10.18632/oncotarget.204 (PMC3100177; doi:10.18632/oncotarget.204)
Supplement: Supplemental Figure 1 — Tie2-mediated modulation of adhesive molecules in gliomas at protein levels. Western blotting analysis of U-87 MG cells treated with Ang1 or vehicle and of U251. vector and U251. Tie2 showing the expression level of N-cadherin and Integrin β1. Tubulin is shown as loading control. [file oncotarget-01-700-s001.ppt]

## Slide 1
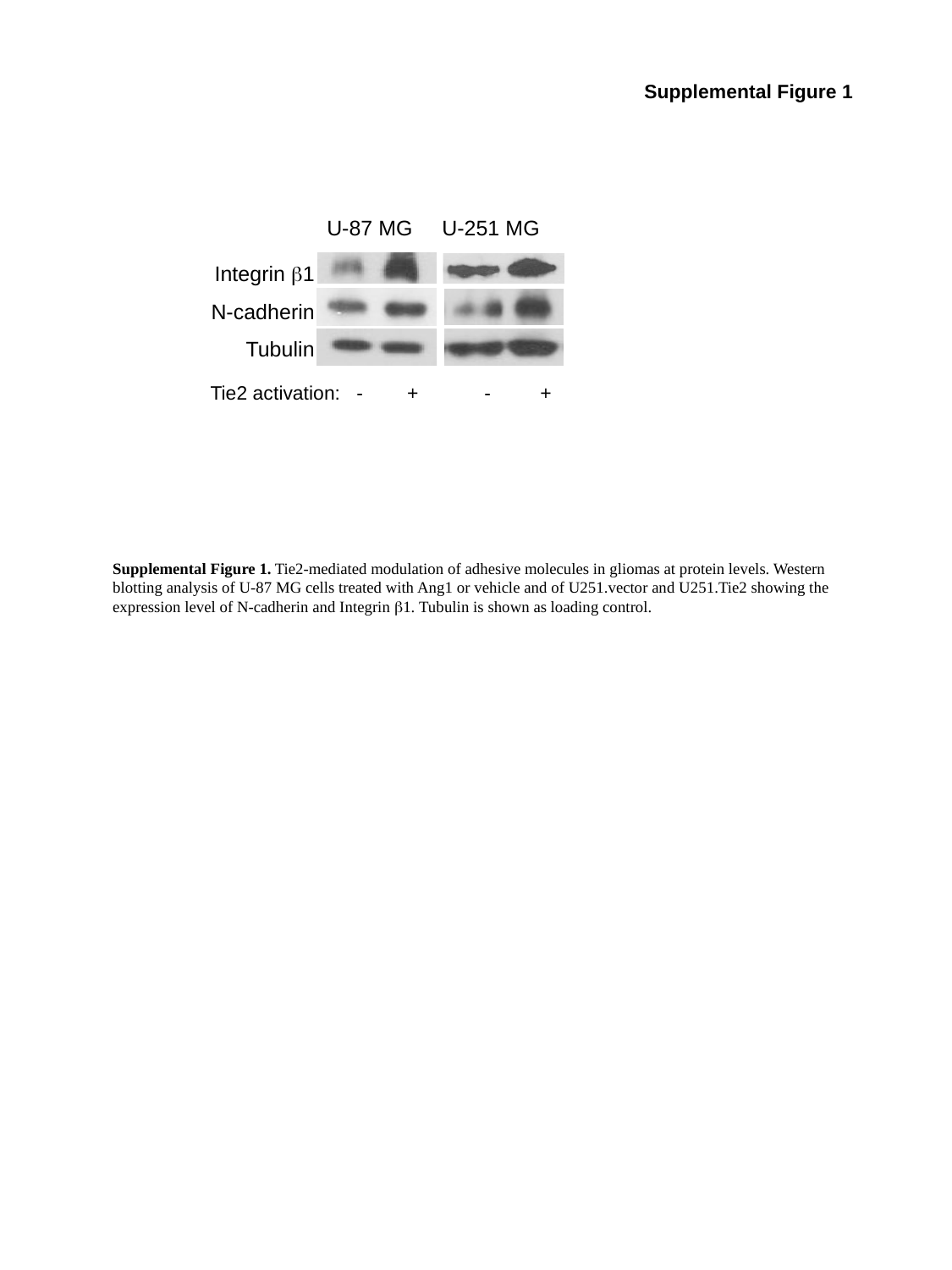

Supplemental Figure 1
U-87 MG U-251 MG
Integrin β1
N-cadherin
Tubulin
Tie2 activation: - + - +
Supplemental Figure 1. Tie2-mediated modulation of adhesive molecules in gliomas at protein levels. Western blotting analysis of U-87 MG cells treated with Ang1 or vehicle and of U251.vector and U251.Tie2 showing the expression level of N-cadherin and Integrin β1. Tubulin is shown as loading control.
