# Supplementary material for: Tie2/TEK Modulates the Interaction of Glioma and Brain Tumor Stem Cells with Endothelial Cells and Promotes an Invasive Phenotype
Source: Oncotarget. 2010 Dec 30;1(8):700–9. doi: 10.18632/oncotarget.204 (PMC3100177; doi:10.18632/oncotarget.204)
Supplement: Supplemental Figure 2 — Tie2-mediated modulation of adhesive molecules in gliomas at transcriptional levels. qPCR analysis of (A) U251.vector and U251.Tie2 and (B) U-87 MG cells treated with Ang1 or vehicle showing the expression level of N-cadherin, α-catenin, and β-catenin. Results represent ΔCT levels (mean ± SEM) over endogenous control GAPDH. [file oncotarget-01-700-s002.ppt]

## Slide 1
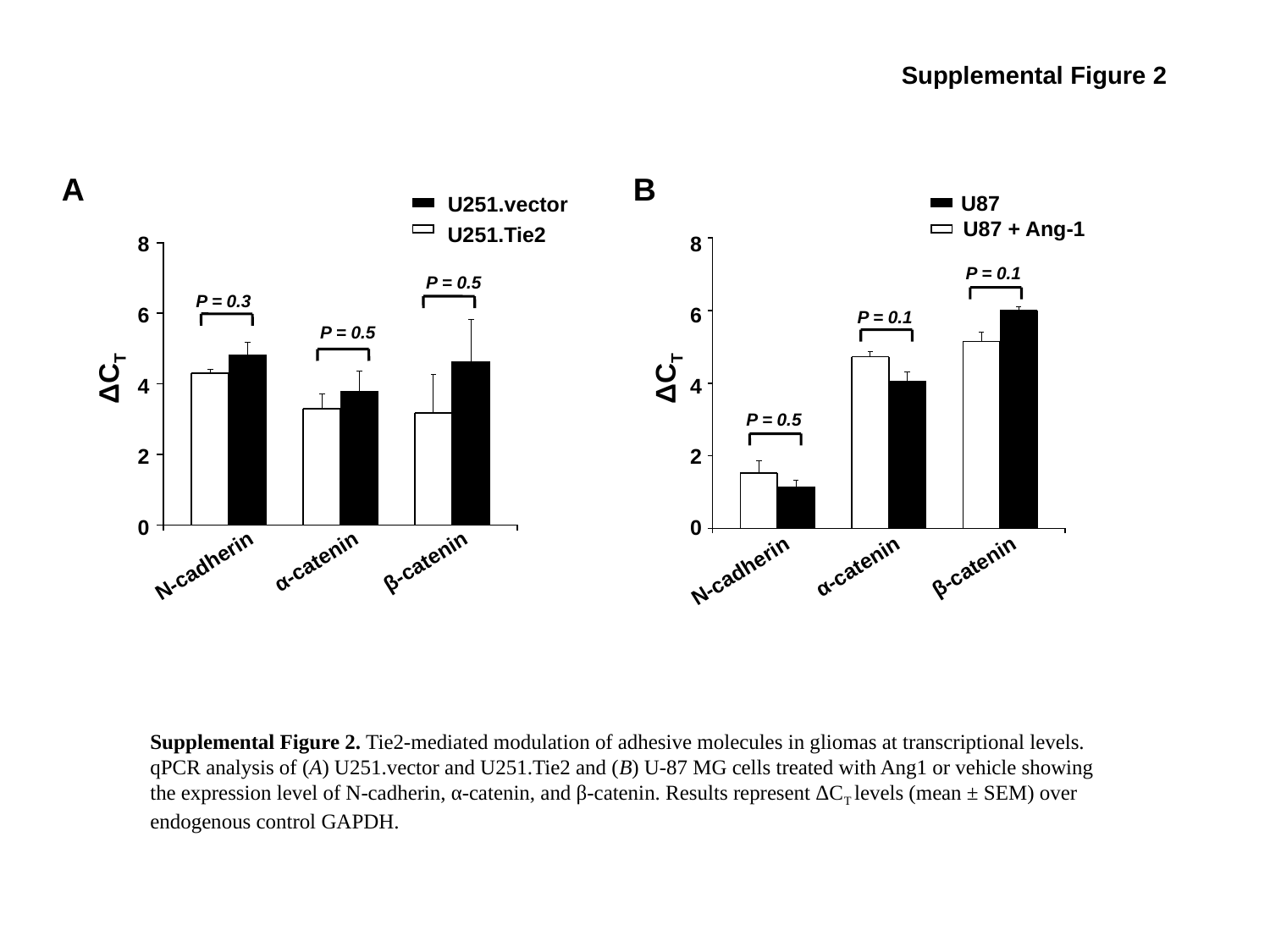

Supplemental Figure 2
A
B
U87
U251.vector
U87 + Ang-1
8
6
4
2
0
8
6
4
2
0
U251.Tie2
P = 0.1
P = 0.5
P = 0.3
P = 0.1
P = 0.5
ΔCT
ΔCT
P = 0.5
α-catenin
β-catenin
α-catenin
β-catenin
N-cadherin
N-cadherin
Supplemental Figure 2. Tie2-mediated modulation of adhesive molecules in gliomas at transcriptional levels. qPCR analysis of (A) U251.vector and U251.Tie2 and (B) U-87 MG cells treated with Ang1 or vehicle showing the expression level of N-cadherin, α-catenin, and β-catenin. Results represent ΔCT levels (mean ± SEM) over endogenous control GAPDH.
| |
| --- |
